# HIN1/353: Speculum: An Attempt to Establish a Broad Communication between Otolaryngologists

**DOI:** 10.2196/jmir.1.suppl1.e32

**Published:** 1999-09-19

**Authors:** F Wallner

**Affiliations:** ^1^Universitäts-HNO-Klinik, HeidelbergGermany

**Keywords:** Communication, Clinical Information Web Site

## Abstract

**Introduction:**

The health service in Germany is traditionally divided into the areas of the ambulatory and stationary attendance of patients, as well as the research and teaching. Communication between these areas is usually limited to letters, meetings, congresses and journals. The rapid extension of the Internet in the last years serves the opportunity of new communication pathways between hospitals, medical practices and searching departments - and to the patient.

**Methods:**

By providing the Web site of the Department of Otolaryngology, the University Hospital of Heidelberg, we have tried to supply, as much as possible, communication opportunities. Besides the usual information about hospital and physicians, there are reports on research projects of individual clinical departments, on scientific publications and congresses. In addition we offer a forum for the discussion of clinical or scientific questions. Assistant medical directors and scientists of our hospital participate in this forum. Furthermore, for general comments and topics there exists a guest book. For regular information about news there is an e-mail newsletter. The homepage has been announced using Internet directories and local meetings.

**Results:**

The supply was assumed good in principle, in the guest book there were almost exclusively positive expressions about our homepage. The project was presented further in a magazine for established ENT physicians. However, more patients rather than our physician colleagues placed questions. The maintenance and the regular updating of the pages are more difficult and complex compared to what was anticipated at first. The latter problem prevented us to always keep the newest status of information on our pages.

**Discussion:**

The frequency of hits by our visitors shows that we are on the correct way. However, the supply is still by far not assumed in the level that we would require it to be. This can be explained by the following two reasons. On the one hand our page could be further improved to include more services, while it remains easy-to-use. On the other hand, not every colleague is familiar with the new media and available possibilities. It is anticipated that the latter will change in the future. In the new era of the networked healthcare, it becomes critical that such services, such as the one described in our paper, should ensure reliability, quality of content and cost-effectiveness.

